# Structural characterization of strawberry pomace

**DOI:** 10.1016/j.heliyon.2024.e29787

**Published:** 2024-04-17

**Authors:** Arland T. Hotchkiss, Hoa K. Chau, Gary D. Strahan, Alberto Nuñez, Andrew Harron, Stefanie Simon, Andre K. White, Senghane Dieng, Eugene R. Heuberger, Ian Black, Madhav P. Yadav, Marjorie A. Welchoff, Julie Hirsch

**Affiliations:** a Dairy & Functional Foods Research Unit, U.S. Department of Agriculture^1^, 600 E. Mermaid Lane, Wyndmoor, PA, 19038, USA; b Sustainable Biofuels and Co-Products Research Unit, Agricultural Research Service, U.S. Department of Agriculture, 600 East Mermaid Lane, Wyndmoor, PA, 19038, USA; c Ingredion, Inc., 10 Finderne Avenue, Bridgewater, NJ, 08807, USA; d Kerr by Ingredion, 2340 Hyacinth Street NE, Salem, OR, 97301, USA; e Complex Carbohydrate Research Center, The University of Georgia, 315 Riverbend Road, Athens, GA, 30602, USA; f Digestiva, Inc., 2860 Covell Blvd., Davis, CA, 95616, USA

**Keywords:** Strawberry, Xyloglucan, Rhamnogalacturonan, Beta-glucan, Dietary fiber, Oligosaccharide

## Abstract

Strawberries are a nutrient dense food rich in vitamins, minerals, non-nutrient antioxidant phenolics, and fibers. Strawberry fiber bioactive structures are not well characterized and limited information is available about the interaction between strawberry fiber and phenolics. Therefore, we analyzed commercial strawberry pomace in order to provide a detailed carbohydrate structural characterization, and to associate structures with functions. The pomace fraction, which remained after strawberry commercial juice extraction, contained mostly insoluble (49.1 % vs. 5.6 % soluble dietary fiber) dietary fiber, with pectin, xyloglucan, xylan, β-glucan and glucomannan polysaccharides; glucose, fructose, xylose, arabinose, galactose, fucose and galacturonic acid free carbohydrates; protein (15.6 %), fat (8.34 %), and pelargonidin 3-glucoside (562 μg/g). Oligosaccharides from fucogalacto-xyloglucan, methyl-esterified rhamnogalacturonan I with branched arabinogalacto-side chains, rhamnogalacturonan II, homogalacturonan and β-glucan were detected by MALDI-TOF MS, NMR and glycosyl-linkage analysis. Previous reports suggest that these oligosaccharide and polysaccharide structures have prebiotic, bacterial pathogen anti-adhesion, and cholesterol-lowering activity, while anthocyanins are well-known antioxidants. A strawberry pomace microwave acid-extracted (10 min, 80 °C) fraction had high molar mass (2376 kDa) and viscosity (3.75 dL/g), with an extended rod shape. A random coil shape, that was reported previously to bind to phenolic compounds, was observed for other strawberry microwave-extracted fractions. These strawberry fiber structural details suggest that they can thicken foods, while the polysaccharide and polyphenol interaction indicates great potential as a multiple-function bioactive food ingredient important for gut and metabolic health.

## Highlights

• Strawberry insoluble dietary fiber contained both rhamnogalacturonan I and II.
• Fucogalacto-xyloglucan, β-glucan and pelargonidin 3-glucoside were also found.
• Polysaccharide random coil shape may be responsible for polyphenol binding.
• Commercial strawberry pomace can be a functional food ingredient thickening agent.

## 1 Introduction

Strawberry is a popular fruit due to its flavor, fragrance, texture, color, nutritional,antioxidant, immunostimulatory, prebiotic and gut-brain axis properties derived from vitamins, minerals, polyphenols, anthocyanins, ellagitannins, and dietary fiber [[1], [2], [3], [4], [5], [6]]. There is substantial evidence that consumption of dietary fiber reduces the risk of cardiovascular disease, type 2 diabetes, and some types of cancer. The beneficial physiological effects of dietary fiber start in the gut. Therefore, the more that we know about fiber composition and physical properties, the better that we can understand the role of the gut microbiota in health outcomes. The gut microbial composition depends on these complex dietary components that are not digested and provide substrates for fermentation by health-promoting probiotic bacteria or those microbes that can do harm [7].

Unfortunately, Americans consume only 58 percent of the recommended dietary fiber amount (30g per day), and low intake is a public health concern for the general U.S. population [[8], [9], [10]]. Fruits, including strawberries, and vegetables, are rich sources of both soluble and insoluble indigestible fiber carbohydrates [[11], [12], [13], [14]]. In addition to insoluble fractions, there are a wide range of complex ‘fibrous’ carbohydrate structures and complexes, with interactions between plant cell wall cellulose, hemicellulose, pectin, protein, lignin and lipids. Due to non-climacteric physiology, strawberries must be picked ripe with a limited time before they soften due to microbial or native enzymatic degradation of pectin in the plant cell walls [3,5,15,16]. Strawberry is a model system for plant cell wall dietary fiber structure during ripening with arabinose release observed during softening [3,16]. The purpose of this work is to provide a deeper understanding of carbohydrate structures and complexes derived from strawberry processing by-products, which could serve as a delicious source of fiber in foods [11,17].

In addition to health benefits, insoluble fiber also contributes to hydrocolloid physical functionality in food. Pectin is traditionally known as a soluble fiber hydrocolloid that provides viscosity and gel-forming properties [18,19]. Previously, we reported that pectin-rich, largely insoluble citrus fiber had high water-holding capacity and fat-replacement properties [20] and that rhamnogalacturonan-rich insoluble dietary fibers from blueberry, cranberry, red beet and carrot had composition that suggests prebiotic and anti-viral properties [[21], [22], [23], [24]]. Therefore, both soluble and insoluble dietary fiber are responsible for important hydrocolloid and health beneficial properties.

Hydrocolloid and bioactive properties are determined by plant cell wall composition and structure [14,25]. Soluble fiber networks surround cellulose microfibrils in plant cell walls, with molecular weight, side chains, the type and amount of branching, charge, and hydrophobicity all playing a role for each of the components [26]. These fiber polysaccharides include homogalacturonan (HG), rhamnogalacturonan I (RG-I), rhamnogalacturonan II (RG-II) and xylogalacturonan (XGA) that are pectin. The RG-I is branched with arabinogalactan side chains substituted on rhamnose residues, while RG-II has a HG backbone substituted with complex branched side chains [14,27]. Homogalacturonan consists of an α-(1–4)-d-galacturonic acid polymer that is partially methyl-esterified at the O6 position. Xylogalacturonan has a HG backbone with galacturonic acid substituted at the O3 position with single xylose residues in plant reproductive tissue [27]. Pectin gelling properties are determined by the degree of esterification (DE), either with sucrose added (DE > 50 %), or in the presence of divalent cations (DE < 50 %). The relative proportion of HG and branched RG-I also contributes to the gel strength. Types of hemicellulose include xyloglucan, glucurono-arabinoxylan or β-glucan depending on the plant source. Dicotyledonous plants and some monocots produce xyloglucan consisting of a β-(1–4)-glucan backbone with xylose attached to O6 of glucose, and fucogalacto- or arabinose substituents attached to the O2 of xylose depending on the plant source. A single letter code was developed to describe the diversity of xyloglucan side chains [28,29]. Some monocots produce glucurono-arabinoxylan that consists of a β-(1–4)-xylan backbone with single arabinose and glucuronic acid substituents. A third hemicellulose β-glucan, is produced in the plant order Poales, consisting of cello-triose and cello-tetraose connected by β-(1–3)-glucosyl-linkages. Glucomannan and galacto-glucomannan and galactomannan are additional less abundant hemicellulose structures found in some plants [14]. Finally, cellulose is β-(1–4)-glucose parallel chains hydrogen-bonded to each other in a crystalline insoluble structure (14). Cell wall proteins, including hydroxy-proline-rich extensin [30] and arabinogalactan proteins [31], associated with pectin, were reported to be responsible for pectin emulsifying properties [32].

The plant cell wall extraction method (acid-base, alcohol precipitation, enzymatic hydrolysis, β-elimination, ultrasonic, microwave-assisted extraction, or by gel column chromatography) also impacts plant cell wall polysaccharide hydrocolloid composition, structure, and functionality. Microwave-assisted extraction takes minutes rather than the hours necessary for conventional commercial procedures, and reduces degradation by hot acidic conditions that may alter the composition critical for bioactive and hydrocolloid properties [[33], [34], [35], [36], [37], [38]]. Commercial production of fruit, vegetable, and grain processing fiber-rich side-streams offer nutritious, bioactive, sustainable, affordable, plant-based and clean label ingredients for the next generation of new food products that are desirable by consumers [[39], [40], [41]]. In addition to strawberry fiber structural details, we would like to understand the unexplored mechanism by which fiber provides nutritional benefits. In this paper, we characterized and compared the dietary fiber in the strawberry pomace remaining after commercial processing that included enzymatic treatment for juice clarification. Pomaces from the fruit and vegetable industry are considered potential food ingredients with promising functionalities, and strawberry pomace is a rich source of flavonoid and anthocyanin phenolics with antioxidant, α-glucosidase and anti-proliferative properties [42,43]. Our research serves to provide comprehensive, detailed carbohydrate composition and structural analysis of strawberry pomace and extracted fractions that may predict function.

## 2 Materials and methods

### 2.1 Chemicals

Shodex Pullulan-P82 standard set was purchased from JM Science (Grand Island, NY). Bovine serum albumin (ca. 98 % monomer) was purchased from Sigma-Aldrich Co. (St. Louis, MO. Sodium nitrate (NaNO_3_), reagent grade, and sodium azide (NaN_3_) were purchased from Sigma Chemical Co. (St. Louis, MO). A 0.05 M NaNO_3_ HPLC mobile phase, containing 0.01 % (m/v) NaN_3_ as a preservative, was prepared in de-ionized water. A 12.1 M HCl was used to prepare the HCl solutions (Fisher Scientific, Fairlawn, NJ). Deionized water was used to prepare all solutions and a 0.45 μm filter was used to filter the solutions (Millex-HV, PVDF, Millipore Corp., Billerica, MA).

### 2.2 Preparation of materials

Strawberry (*Fragaria ananassa* cv. Elsanta) fractions were obtained from Kerr by Ingredion. The strawberries were harvested during the 2019 growing year in California, between mid-June and mid-July. The frozen fruit was shredded, heated to 51.7 °C, then passed through a comminutor with a ¼” screen. The strawberries were treated at 54 °C at the unbuffered strawberry native pH (3.5) with polygalacturonase (190 ppm; DSM, Amsterdam, the Netherlands), arabinase (50 ppm; Takabio, Shin Nihon Chemical Co., Anjo, Japan), and a cellulase (40 ppm; β-(1–4)-d-glucanase, Novozymes, Bagsværd, Denmark) enzymes together at the same time. The enzyme-treated mash was held at 48.9 °C for approximately 2 h, before being passed through a decanter centrifuge (9345 kg/h, GEA, Dusseldorf, Germany) without pasteurization or other heating to inactivate enzymes. The mash at this stage was referred to as the pomace fraction (PF) and was kept frozen (−20 °C) until freeze drying for further studies.

The PF was fractionated in water by suspending 250 mg of PF in deionized (DI) water to produce a 1 % (m/v) suspension. The suspension was stirred at room temperature for 24 h and then centrifuged at 26,000×*g* at 20 °C for 30 min. The supernatant was removed and filtered through a 0.45 μm, 33 mm syringe filter and then the solution was lyophilized to produce a freeze-dried water-soluble fraction (WSF). The water-insoluble fraction (WIF) was collected, washed with DI water using a vacuum filtration funnel and No. 1 filter paper, and lyophilized for further analysis.

Further fractionation of strawberry pomace was conducted using microwave-assisted acid extraction with a MarsX model MDS-2000 microwave (CEM Corp., Matthews, NC). One gram of dried strawberry PF was microwave extracted in 25 mL of HCl with various conditions (pH 1, 3 min, 120 °C; pH 2, 3 and 6 min, 120 °C, 10 min, 80 °C) [22]. After cooling in an ice bath for 30 min, the extracted samples were filtered through miracloth. The solubilized supernatant was precipitated with a 1:2 ratio to 95 % isopropyl alcohol (IPA) (v/v) at room temperature for 30 min, filtered with miracloth, followed by washing twice with 95 % IPA (v/v) and once with 100 % IPA [22]. For the microwave extraction (MWE) at pH 1.0 for 3 min at 120 °C, the precipitate was collected, stored at 4 °C overnight, filtered then dried in a vacuum overnight. For the MWE at pH 2 for 3 and 6 min at 120 °C, and 10 min at 80 °C, no precipitate formed after 30 min, so an additional 200 mL of 95 % IPA was added and stored at 4 °C overnight, filtered, followed by washing twice with 95 % IPA (v/v) and once with 100 % IPA prior to centrifugation and drying. This fraction represented fraction one (Frac 1) of the 6 min, 120 °C microwave-extracted fraction. An additional 200 mL of 100 % IPA was added to the wash liquid of the 6 min, 120 °C fraction and then stored overnight at 4 °C to produce a second fraction (Frac 2) of the 6 min, 120 °C fraction. The vacuum-dried MWE samples were stored in a freezer (−20 °C) until further characterization.

### 2.3 Moisture, protein, ash, and fat

Moisture, protein (N × 6.25) and ash contents of all samples were determined using AACC Approved Methods 44-19, 46-30 and 08-01 respectively as reported previously [21]. Crude fat was also determined following pomace hydrolysis using AOAC method 954.02 by Eurofins (Des Moines, IA). The values are reported as means (± standard deviation) of triplicate analysis.

### 2.4 Dietary fiber

Soluble dietary fiber (SDF), insoluble dietary fiber (IDF) and total dietary fiber (TDF) of samples were determined using AOAC 991.43, AACC 32–07.01, NMKL 129,2003 methods on the ANKOM A2000 Dietary Fiber Analyzer (ANKOM Technology, Macedon, NY). The values reported are means (± standard deviation) of duplicate analysis.

### 2.5 Monosaccharide analysis

Monosaccharides were analyzed by High Performance Anion Exchange Chromatography and Pulsed Amperometric Detection (HPAEC-PAD) using a CarboPac PA-20 column (Thermo Fisher Scientific, Waltham, MA) following methanolysis according to Zhao et al. [44] as reported previously [21]. Major monosaccharides and disaccharides were also determined using HPAEC-PAD without methanolysis [21].

### 2.6 Oligosaccharide structure

Oligosaccharide structure was determined by Matrix-Assisted Laser Desorption/Ionization Mass Spectrometry with automated tandem Time of Flight (MALDI-TOF/TOF MS) fragmentation of selected ions of strawberry fractions using a 4700 Proteomics Analyzer mass spectrometer (Sciex, Framingham, MA) in the positive reflectron mode. Samples containing oligosaccharides (3–5 mg) were dissolved in 1 mL of water and cleaned with graphitized carbon tips (NuTip 10–200 μL, Glygen Co. Columbia, MD). The tips were first conditioned by passing 100 μL of acetonitrile:water (50:50) and then washed 4 times with 100 μL of water. After conditioning, 100 μL of the oligosaccharide solution was loaded on the tip, washed 3 times with 100 μL of water and the oligosaccharides were eluted with 100 μL of acetonitrile:water (30:70) 0.1 % TFA (v/v). The extracted solution was dried in a vacuum centrifuge, resuspended in 2,5-dihydroxy-benzoic acid (10 mg/mL in acetonitrile:water (50:50), 0.1 % TFA (v/v)) as the matrix, prior to spotting on a MALDI plate [21].

### 2.7 Oligosaccharide and polysaccharide glycosyl-linkage positions

Glycosyl linkage analysis was performed by combined gas chromatography/mass spectrometry (GC/MS) of the partially methylated alditol acetates (PMAAs) derivatives produced from strawberry fractions as detailed by Black et al. [45]. In order to improve sample solubility and methylation, the samples were homogenized in 2-mL reinforced microtubes containing ceramic beads (Omni International), using a bead ruptor. The homogenized sample was then dissolved in 1-ethyl-3-methylimidazolium acetate (EMIMAc) overnight. The sample was then acetylated and dialyzed. The dried sample was then methylated with dimsyl anion, reduced with lithium aluminum deuteride, and dialyzed again. The dried, reduced sample was subjected to a second round of methylation and hydrolyzed. The anomeric carbons were then reduced, and finally the free hydroxyls were acetylated. A detailed procedure was published previously by Black et al. [45]. The resulting PMAAs were analyzed on an Agilent 7890A GC interfaced to a 5975C MSD (mass selective detector, electron impact ionization mode); separation was performed on a 30 m Supelco SP-2331 bonded phase fused silica capillary column.

### 2.8 Anthocyanins

Strawberry PF pomace was analyzed by Eurofins (Des Moines, IA) using reversed phase gradient HPLC with a C18 column, a diode array detector (520 nm) and anthocyanin standards according to Durst & Worsted [46]. The values were reported as means (± standard deviation) of triplicate analysis.

### 2.9 High Performance Size Exclusion Chromatography analysis of macromolecular molar mass, viscosity, size and shape distribution

High Performance Size Exclusion Chromatography (HPSEC) was used to analyze strawberry PF, WSF, WIF and fractions microwave-extracted at various pH values, times, and temperatures as reported previously [22]. The dn/dc values used were 0.132, 0.148 and 0.185 (mL/g), for strawberry samples, pullulan and bovine serum albumin standards, respectively. The values were reported as means (± standard deviation) of triplicate analysis.

### 2.10 NMR analysis of molecular fine structures

Strawberry pulp (PF), 6/120 min/°C, pH 2, 2nd fraction prepared in 2019 and the 3/120 min/°C, pH 1 microwave-extracted fraction prepared in 2023 were dissolved in D_2_O with the addition of d_4_-trimethylsilylpropanoic acid (TMSP) as an internal chemical shift reference and NaN_3_. All spectra were measured at either 40 °C or 75 °C on a 14 T Agilent DD2 NMR spectrometer (Santa Clara, CA) using a 5 mm OneNMR probe with z-axis pulsed field gradients. Experimental parameters and analysis methods were largely the same as previously reported [24]. The 1D-^1^H NMR spectra (600 MHz) were acquired using the WET water suppression, a 45° pulse angle, a relaxation delay of 1.5, 2 or 5 s and spectral widths of 6.5–10 ppm, using 32k points and averaged over 16–64 transients. The 1D-^13^C spectra (150 MHz) were acquired semi-quantitatively using a 45° pulse angle, relaxation delay of 1–3 s, acquisition time of 0.87 s, spectral width of 250 ppm, 32k data points which were averaged over 56,000–200,000 transients, and employed proton decoupling during only the acquisition period. The T1 relaxation times were not measured. The two-dimensional, homonuclear COSY and zTOCSY experiment were gradient-enhanced and acquired with spectral widths of 6.7 or 10 ppm in both dimensions, using 2K or 4K points in the directly-detected dimension, averaged over 32 transients per acquisition, and 256 or 512 indirectly-detected increments. The zTOCSY experiments were acquired using 80, 120, 150 and 22 ms mixing times. Gradient versions of the HSQC (multiplicity-edited), HSQC-TOCSY, HMBC and H2BC heteronuclear experiments were acquired with either 2k or 4k directly-detected data points, and spectral widths of either 6.5 or 10 ppm in the directly-detected (^1^H) dimension. Various spectral widths were used in their indirectly-detected dimensions. The HSQC and HSQC-TOCSY widths were 130 ppm, the HMBC widths were 211 ppm, and the H2BC widths were 85 ppm. The signals of most spectra were averaged over 64–128 transients per increment. The indirect dimensions were detected in 300–400 increments, except for the constant time H2BC, which used 128–160 increments. The HSQC-TOCSY experiments were measured using 80–150 ms mixing times. Multiple C–H coupling constants were used in HMBC experiments (5, 8, 12 and 16 Hz). All NMR spectra were processed using OpenVNMRJ [47] and visualized using UCSF Sparky software [48].

## 3 Results and discussion

### 3.1 General composition

The strawberry pomace protein, ash, fat, and moisture content is listed in Table 1. Strawberry PF was rich in protein (15 %), moisture (10 %), with lesser amounts of fat (8 %) and ash (3 %) (Table 1). Pukalskiene et al. [43] reported that strawberry pomace contained 13.3 % protein, 12.03 % fat, 5.6 % moisture and 5.3 % ash with the amount of fat determined by the seed content and the ash content varying with the harvest season. The strawberry fiber protein content that we observed in PF (15 %) and WIF (13 %) was higher than what we observed for blueberry, red beet and carrot fiber as well as sugar beet pectin [21,22,24,35]. Raw strawberries contain 0.67 % protein [49], which supports a protein-fiber association in these fruits as reported earlier [50]. The strawberry PF fat content (8 %) was the highest compared to blueberry, red beet and carrot fiber [[21], [22], [24]]. Strawberry PF moisture and ash levels were in between those values that we observed previously for blueberry and red beet fiber [[21], [22]].

**Table 1:** Composition of strawberry pomace fractions (PF, WSF, WIF).

%	PF	WSF	WIF
Protein	15.6 ± 2.7	0.7 ± 0.0	13.6 ± 1.4
Moisture	10.5 ± 1.1	33.1 ± 0.5	7.8 ± 0.2
Total Ash	3.3 ± 0.3	4.4 ± 0.3	2.1 ± 0.1
Total Fat	8.3 ± 1.4	ND	ND

Means (± standard deviation) of pomace fraction (PF), water-soluble fraction (WSF), and water-insoluble fraction (WIF) means are expressed as percentages (% (mass/volume)). ND = not determined.

### 3.2 Dietary fiber

**Fig. 1:**
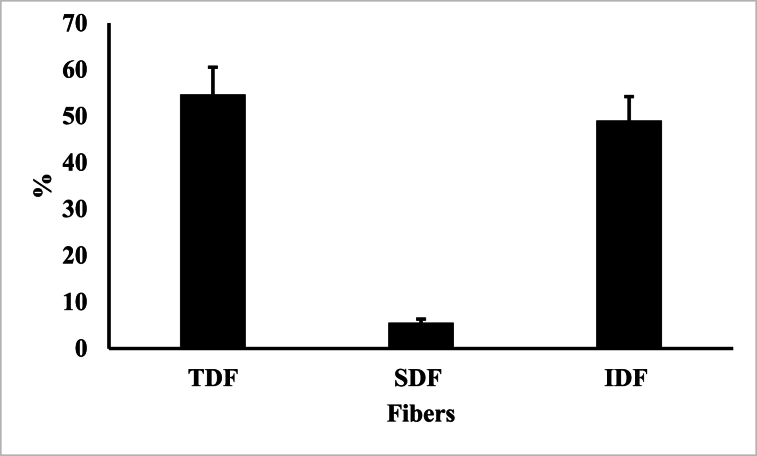
Dietary fiber analysis of strawberry pomace fraction PF. Total dietary fiber (TDF), soluble dietary fiber (SDF) and insoluble dietary fiber (IDF) weight percent (%) mean (± standard deviation) values of duplicate analysis.

Strawberry PF is also a rich source of total dietary fiber (54.7 %) that was mostly insoluble dietary fiber (49.1 %) (Fig. 1) like what we observed with blueberry, red beet and carrot fiber [21,22,24]. When the strawberry water-soluble fraction (WSF) was removed from the PF to produce WIF, it is interesting to note that the WIF contained only 4.5 % soluble dietary fiber (SDF) (Fig. S1), which was the lowest SDF in WIF that we observed compared to blueberry (13.5 %) [21] and red beet (9.8 %) [22] WIF. Raw strawberry fruit was also rich in dietary fiber that was mostly insoluble fiber (61 %), while fresh blueberry fruit also was rich in insoluble dietary fiber (79 %) [51]. Therefore, the enzymatic (polygalacturonase, arabinase and cellulase) treatment of the strawberry fruit mash used to produce PF inflated the mostly insoluble nature of the dietary fiber compared to that reported for fresh fruit, which indicated that the enzymes preferentially degraded the SDF.

### 3.3 Monosaccharide analysis

**Fig. 2:**
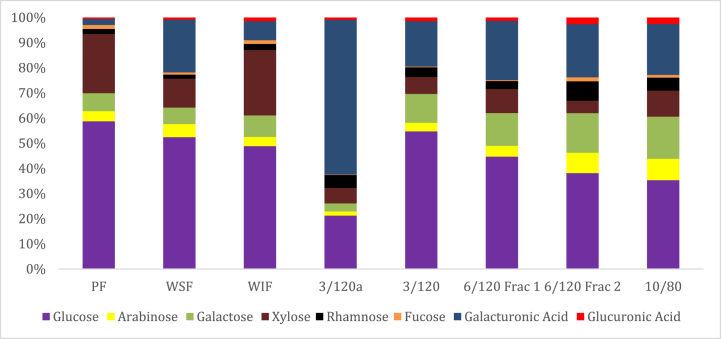
Monosaccharide analysis (mole %) of strawberry pomace fraction (PF), water-soluble fraction (WSF), water-insoluble fraction (WIF) and fractions microwave-extracted for 3 min, 120 °C at pH 1 (3/120a), and the remaining fractions were extracted at pH 2 for 3 min, 120 °C (3/120), 6 min, 120 °C, 1st fraction collected after precipitation (6/120 Frac 1), 6 min, 120 °C, 2nd fraction after precipitation (6/120 Frac 2), and 10 min, 80 °C (10/80).

The strawberry PF contained mostly glucose and xylose (Fig. 2). Free glucose, fructose, xylose, arabinose, galactose, fucose and galacturonic acid were also detected in the strawberry PF using HPAEC-PAD without methanolysis. These free carbohydrates probably make up the remaining 7.6 % of the strawberry PF (Table 1 protein, moisture, ash, fat + dietary fiber = 92.4 %). The strawberry microwave-extracted fractions contained mostly glucose and were enriched in galacturonic acid and galactose (Fig. 2). Galacturonic acid, galactose, and arabinose, with lower amounts of rhamnose, xylose, glucose, fucose, mannose, and glucuronic acid were reported in strawberry pectin previously [3,5,15,25,50]. The monosaccharide composition of strawberry microwave-extracted fractions was consistent with a rhamnogalacturonan I structure (Fig. 2), which was reported previously in strawberry fractions [[3], [5], [50], [52]]. More strawberry homogalacturonan than rhamnogalacturonan I was reported based on monosaccharide values in organic and conventional strawberries with homogalacturonan values generally decreasing during cold storage due to microbial enzymatic activity [5]. Our enzymatic treatment of the strawberry fruit released free sugars, reduced arabinose and galacturonic acid levels, and enriched monosaccharides consistent with xyloglucan structure (Fig. 2). Xyloglucan was previously reported in strawberry fractions based on monosaccharide ratios [50], HPAEC-PAD analysis [53] and LM15 (XXXG specificity) [29] and LM25 (XXLG, XLLG specificity) antibody epitope mapping [54].

### 3.4 Oligosaccharide structure

**Fig. 3:**
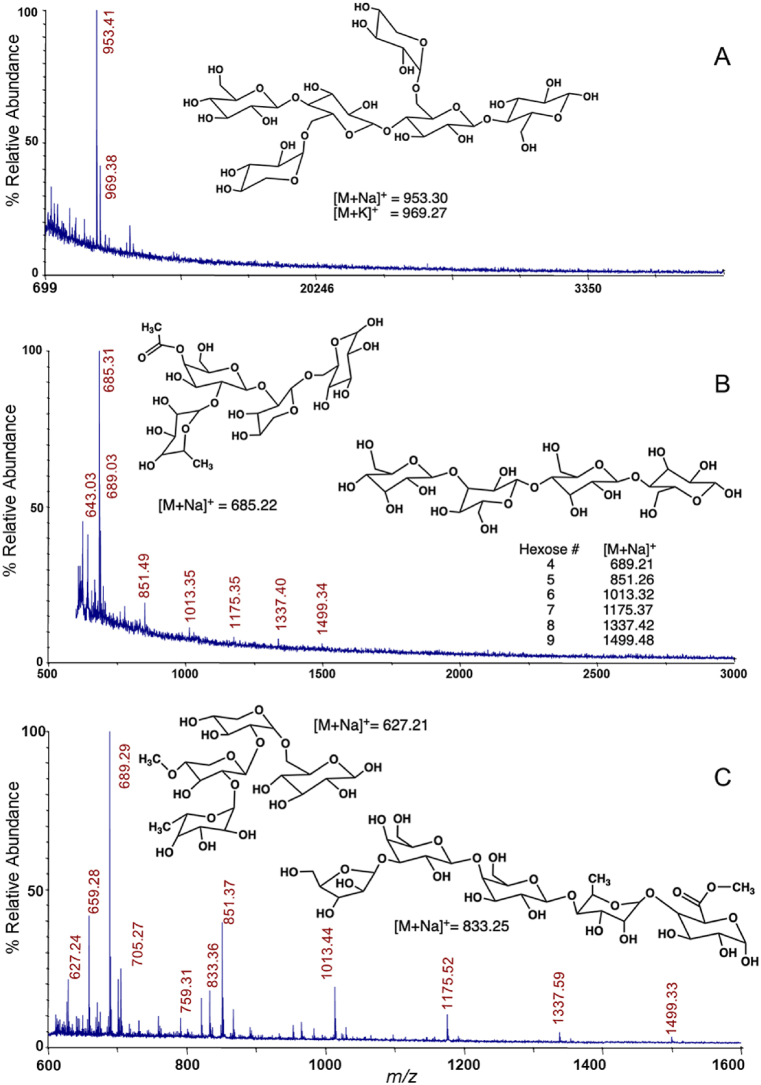
Matrix-Assisted Laser Desorption/Ionization - Time of Flight Mass Spectrometry of the strawberry PF (A), microwave-extracted fraction (3/120 min/°C, pH 1) (B), and the residue remaining after microwave extraction (3/120 min/°C, pH 1) (C). The mass/charge (*m*/*z*) is shown in the positive reflectron mode.

Oligosaccharide structures that bound to graphitized carbon and were eluted with acetonitrile:water (30:70), 0.1 % TFA (v/v) [21] were analyzed by MALDI-TOF MS (Fig. 3, Table 2). The major strawberry PF oligosaccharide structure detected was a xyloglucan hexasaccharide GXXG (*m*/*z* = 953, Fig. 3A), while the major oligosaccharide present in the 3/120 min/°C, pH 1 fraction was a xyloglucan tetrasaccharide F (*m*/*z* = 685, Fig. 3B), and another xyloglucan tetrasaccharide methylated E was observed in the residue remaining after the 3/120 min/°C, pH 1 microwave extraction (*m*/*z* = 627, Fig. 3C). The *m*/*z* = 685 F tetrasaccharide is drawn with galactose acetylation at O-4 based on linkage analysis data (Table 3). Sycamore xyloglucan F had galactose acetylation at O-6 and O-3 galactose acetylation has also been reported [55]. The unacetylated *m*/*z* = 643 xyloglucan F tetrasaccharide was also detected in the strawberry 3/120 min/°C, pH 1 fraction. The *m*/*z* = 953 xyloglucan hexasaccharide was observed in cranberry with SGGG structure based on MALDI-TOF/TOF MS/MS and 2D-NMR [56]. Cranberry is a member of the Ericales which produces both fucogalacto-xyloglucan and arabino-xyloglucan [[21], [29], [56], [57]], while strawberry is a member of the Rosales with only fucogalacto-xyloglucan structure reported previously in strawberry (XXXG, XXFG) [53] and apple (XXXG, XXFG, XLFG) [58]. The GXXG xyloglucan is the first report of this structure in strawberry, but it was reported previously in sweet pepper, a member of the Solanales [57]. Other strawberry xyloglucan oligosaccharides similar to the GXXG structure (*m*/*z* = 629, 659, 791, 1115) were also detected (Table 2).

**Table 2:** Matrix-Assisted Laser Desorption/Ionization - Time of Flight Mass Spectrometry analysis of strawberry fractions.

*m/z*^a^	Structure^b^	Xyloglucan	*m/z*^a^	Structure^b^	Xyloglucan
627	Pent2dHexHexMe	E^c^	1097	HexA6 or Pent3dHexHex2HexAMe	
629	Pent2Hex2	XX	1115	Pent2Hex5	GXXGG
643	PentdHexHex2	F	1157	PentdHexHex3HexA2 or PentdHexHex4HexAMe	
659	PentHex3	GL	1175/1191	Hex7	
685	PentdHexHex2Ac	F	1229	Pent4dHexHex2HexAMe	
689/705	Hex4		1273	HexA7	
701	dHexHexHexA2		1319	PentdHexHex4HexA2 or PentdHexHex5HexAMe	
745	HexA4		1337	Hex8	
759	Pent3dHexHexMe		1449	HexA8	
791	Pent2Hex3	GXX	1451	Pent2dHexHex4HexA2	
833	PentdHexHex2HexAMe		1493	Pent6dHexHexHexA2 or Pent6dHexHex2HexAMe	
851/867	Hex5		1499	Hex9	
921	HexA5		1555	Pent4Hex6Me	
953/969	Pent2Hex4	GXXG	1597	Pent4Hex6MeAc	
965	Pent2dHexHex2HexAMe		1613	Pent2dHexHex5HexA2	
995	PentdHexHex2HexA2		1625/1641	HexA9	
1013/1029	Hex6		2161	Hex13Me	

a Nominal mass/charge (*m*/*z*) corresponding to the [M+Na]^+^ or [M+K]^+^ adducts.

b Pentose (Pent), hexose (Hex), deoxy-hexose (dHex), hexuronic acid (HexA), ferulic acid (FA), methyl (Me), acetyl (Ac).

c Methylated.

**Table 3:** Glycosyl-linkage analysis of strawberry fractions.

Linkage^a^	PF	3/120a	3/120	6/120 Frac 1	6/120 Frac 2	10/80
t-Rha*p*	0.1	0.8	0.9	1.3	0.6	1.6
t-Ara*f*	0.5	0.1	2.7	1.9	0.9	2.7
t-Fuc*p*	0.6	–	0.5	0.5	0.4	0.6
t-Ara*p*	0.1	–	0.2	0.3	0.1	0.3
t-Xyl*p*	1.7	1.4	1.0	1.4	1.0	1.2
2-Rha*p*	0.1	2.4	3.1	1.8	2.2	2.9
2-Ara*f*	0.1	–	–	–	–	–
t-Man*p*	0.1	2.0	0.6	0.8	0.6	1.1
3-Rha*p*	–	0.2	–	–	–	–
4-Rha*p*	–	–	0.3	–	0.2	0.3
t-Glc*p*	1.8	6.8	4.7	5.2	5.6	4.2
t-Glc*p*A	0.2	1.3	2.2	1.0	0.9	1.2
3-Ara*f*	0.1	0.7	0.6	1.0	0.6	1.4
t-Gal*f*	0.0	–	0.1	0.2	0.6	0.4
t-Gal*p*	1.0	2.8	4.7	2.8	4.6	3.3
t-Gal*p*A	–	1.0	0.7	0.3	0.4	0.6
4-Ara*p* or 5-Ara*f*	0.2	0.3	0.9	0.6	0.4	0.9
3′-Api	–	–	0.2	–	0.1	0.2
4-Xyl*p*	2.9	3.2	1.8	2.7	1.1	4.1
2-Xyl*p*	0.8	0.5	0.3	0.8	0.8	0.7
3,4-Fuc*p*	–	–	0.3	0.3	0.1	0.3
2,3-Rha*p*	–	–	0.3	–	–	–
3-Glc*p*	3.6	6.7	12.7	12.1	10.9	5.7
2,4-Rha*p*	0.6	2.6	6.0	3.5	5.3	2.2
2-Man*p*	0.1	3.2	0.4	0.7	1.7	0.6
2-Glc*p*	0.0	0.2	0.1	–	0.1	–
2-Glc*p*A	0.1	0.1	0.4	–	0.1	–
3-Gal*p*	0.1	1.1	1.9	1.2	1.5	1.1
4-Man*p*	3.2	6.6	2.4	5.5	3.8	9.1
6-Man*p*	–	1.5	0.2	0.3	0.6	–
2-Gal*p*	0.8	–	0.3	0.7	1.0	0.5
2-Gal*p*A	–	–	0.1	–	–	0.1
6-Glc*p*	0.3	1.4	0.8	1.0	0.9	0.9
4-Gal*p*	0.3	1.9	3.4	3.0	3.3	2.9
4-Gal*p*A	0.4	9.6	7.5	2.8	4.6	4.2
4-Glc*p*	67.4	20.1	20.3	26.1	20.1	21.5
2,4-Xyl*p*	0.7	0.2	0.2	0.3	0.2	–
2,3-Man*p*	–	0.9	0.3	0.5	0.6	9.0
6-Gal*p*	0.2	1.6	3.4	2.2	4.3	1.5
3,4-Gal*p*	–	0.4	0.4	0.1	0.2	0.3
3,4-Gal*p*A	–	1.2	1.1	0.1	0.2	0.7
3,4-Glc*p*	1.5	3.3	0.7	2.3	0.4	1.8
2,3-Glc*p*	0.2	0.4	0.3	–	0.1	–
2,4-Man*p*	–	0.3	–	–	0.1	–
2,4-Gal*p*	–	0.4	0.2	–	–	–
2,4-Gal*p*A	–	0.1	0.2	–	–	–
4,6-Man*p*	1.0	1.2	0.7	1.1	0.5	1.6
3,6-Glc*p*	1.2	3.4	2.2	3.5	4.9	1.5
3,6-Man*p*	–	0.5	0.2	0.4	0.8	0.4
2,6-Man*p*	–	1.1	0.2	0.2	0.4	–
4,6-Glc*p*	7.3	4.9	3.0	6.2	7.3	3.6
4,6-Gal*p*	–	0.1	0.1	0.1	0.1	–
3,6-Gal*p*	0.4	1.2	4.2	3.1	4.8	2.6

a Glycosyl-linkage deduced from GC/MS analysis of per-*O*-methylated alditol acetates. Glycosyl-linkage position (numbers), apiose (Api), arabinose (Ara), fructose (Fru), galactose (Gal), galacturonic acid (GalA), glucose (Glc), glucuronic acid (GlcA), fucose (Fuc), mannose (Man), rhamnose (Rha), xylose (Xyl), furanose (*f*), pyranose (*p*), terminal (t) and not detected (−). Relative peak area percentages (%) of per-O-methylated alditol acetates for the pomace fraction (PF), water-soluble fraction (WSF), water-insoluble fraction (WIF), fractions microwave-extracted for 3 min, 120 °C at pH 1 (3/120a), and the remaining fractions were microwave-extracted at pH 2 for 3 min, 120 °C (3/120), 6 min, 120 °C, 1st fraction collected after precipitation (6/120 Frac 1), 2nd fraction collected after precipitation (6/120 Frac 2) and 10 min, 80 °C (10/80).

Hexose oligosaccharides (*m*/*z* = 689/705, 851/867, 1013/1029, 1175/1191, 1337, 1499) were present in the strawberry fractions and the residue remaining following microwave extraction, which were assigned as β-glucan-oligosaccharides based on linkage and NMR analysis (see below). These oligosaccharides remained soluble at a degree of polymerization (DP) of 9, which means they were not cello-oligosaccharides that have low water-solubility above DP 8.

Rhamnogalacturonan I oligosaccharide with arabinogalacto-oligosaccharide side chains (Fig. 3C), free arabinogalacto-oligosaccharides, and homogalacturonan oligosaccharides were also observed in the strawberry WSF (Table 2). Oligogalacturonic acids were previously detected in pectinase-treated strawberry juices [59]. Oligosaccharides with methyl groups were detected but some methylated structures (*m*/*z* = 1097, 1157, 1319, 1493) could not be distinguished from non-methylated structures within the instrumental error. There were fewer strawberry oligosaccharides with acetyl groups compared to what we detected using the same methods for blueberry and cranberry oligosaccharides, yet the number of structures with methyl groups was similar [[21], [56]].

### 3.5 Linkage analysis

Glycosyl-linkage analysis performed on the strawberry fractions demonstrated that 4- linked-glucose and 3-linked-glucose were abundant linkages (Table 3), which indicated that β-glucan might be present. β-glucan is known to have cholesterol-lowering properties [60]. Rhamnogalacturonan I linkages such as 2-Rha, 2,4-Rha and 4-GalA linkages were also detected in the strawberry fractions (Table 3). This pectin was highly branched with 2,4-Rha 1.9–2.4 x more abundant than 2-Rha in MWE 3 min and 6 min pH 2 fractions but branching decreased with the longer microwave extraction time at 10 min (Table 3). The 3 min pH 1 MWE fraction was the least branched with 0.8 x 2,4-Rha compared to 2-Rha linkages (Table 3). The RG-I sidechains consisted of arabino-oligosaccharide (t-Ara, 3-Ara, 5-Ara), galacto-oligosaccharide (t-Gal, 4-Gal, 4,6-Gal), and arabinogalacto-oligosaccharide (t-Ara, 3-Gal, 6-Gal, 3,6-Gal) linkages. The galacto-oligosaccharide side chains appear to be mostly unbranched since only a minor amount of 4,6-Gal was detected (Table 3). The presence of 3′-Api, t-GalA, t-Fuc, 3,4-Fuc, and 2-GlcA (Table 3) demonstrated that strawberry pectin contains minor amounts of RG-II. While Paniagua et al. [61] proposed that strawberry contained RG-II based on AFM images of endo-polygalacturonase-treated strawberry pectin, this is the first report of RG-II linkages in strawberry. The 3,4-GalA along with t-Xyl linkages (Table 3) indicates that minor amounts of xylogalacturonan was present in strawberry pectin with this polysaccharide most abundant in the 3 min MWE time fractions. The combined arabinase and polygalacturonase enzymatic treatment removed some arabino- and arabinogalacto-oligosaccharide side chains from RG I and removed arabino-oligosaccharide branching since 3,5-Ara and 2,3,5-Ara was not detected (Table 3). The RG I with an arabinogalacto-oligosaccharide side chain (Fig. 3C) was detected in the residue remaining after 3/120 min/°C, pH 1 microwave extraction. Therefore, RG I remained insoluble even following hot acid microwave extraction. Rhamnogalacturonan I was reported to have antiviral properties [62] while the arabino- and galacto-oligosaccharide side chains of RG I have prebiotic properties [[63], [64]].

Pectin and β-glucan were not the only linkages present in strawberry microwave-extracted fractions. Xyloglucan linkages (4-Glc, 4,6-Glc, 2-Xyl, 2-Gal, 2,4-Gal and t-Fuc) confirmed that F xyloglucan structure was present in strawberry (Table 3). The 2,4-Gal linkage confirmed the *m*/*z* = 685 F acetylation at the O4 position (Fig. 3, Table 2). The xyloglucan 2-Xyl and 2-Gal linkages had double the abundance of the t-Fuc linkage, which was consistent with an L xyloglucan structure, and confirmed the *m*/*z* = 659 GL structure (Table 2). While 2-Ara was not detected, 3-Ara was, which suggested that the *m*/*z* = 627 methylated E, was a novel xyloglucan structure. Our linkage data provides confirmation of the strawberry xyloglucan that Legentil et al. [50] proposed based on the high content of glucose and xylose in their sequentially extracted strawberry pectin fractions. Xyloglucan associated with pectin has prebiotic and pathogen anti-adhesion properties [23,56,65].

The presence of t-Man, 4-Man, 4,6-Man, t-Gal and 4-Glc indicated that a mannan, galacto-mannan or galacto-glucomannan was present in the strawberry fractions (Table 3). When the HPAEC-PAD monosaccharide analysis mobile phase conditions were used in order to separate mannose from xylose, then 2.7 mol% mannose was detected in the strawberry fractions (data not shown). A strawberry xylan was also present based on the t-Xyl and 4-Xyl linkages, with t-GlcA and t-Araf linkages confirming that it was a glucurono-arabinoxylan (Table 3). The strawberry PF linkage analysis was repeated with a second batch of strawberry pomace produced in 2021 and the results were almost identical to those from the 2019 strawberry PF shown in Table 3.

### 3.6 Anthocyanin content

The strawberry PF anthocyanin content was 636 μg/g with pelargonidin 3-glucoside (562 μg/g) and cyanidin 3-glucoside (27.7 μg/g) the most abundant (Table 4), which was higher anthocyanin content than reported previously in fresh strawberries and pomaces [[1], [4], [42], [43], [66]]. Minor amounts of malvidin 3-glucoside were reported previously in strawberry [67]. Blueberry anthocyanins including malvidin 3-glucoside, strongly bound to the blueberry pomace fiber during juice processing and this binding was presumed to be ionic between the positively charged anthocyanins and negatively charged pectin [21]. The strawberry PF retained some red color and strawberry fragrance even though it was treated with pectinase enzymes during processing. The anthocyanin analysis was repeated with a strawberry pomace PF produced in 2021 with even higher values (795 ± 79.3 μg/g total anthocyanins) than those from the 2019 strawberry pomace PF shown in Table 4. Malvidin 3-glucoside had prebiotic activity for both *Bifidobacterium* spp. and *Lactobacillus*-*Enterococcus* spp. in human fecal fermentations [68]. It remains to be investigated if strawberry PF has prebiotic activity.

**Table 4:** Strawberry pomace anthocyanins (mean ± standard deviation).

Anthocyanin	(μg/g)
Pelargonidin 3-glucoside	562.0 ± 52.3
Cyanidin 3-glucoside	36.0 ± 3.3
Malvidin 3-glucoside	27.7 ± 2.6
Unknown anthocyanidin	10.4 ± 1.0
Total	636.0 ± 59.1

### 3.7 Macromolecular molar mass, viscosity, size and shape distribution by HPSEC/MALLS

**Fig. 4:**
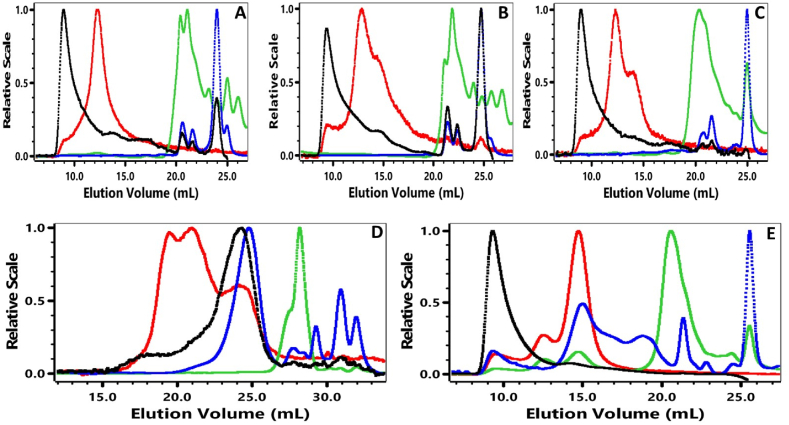
High Performance Size Exclusion Chromatography analysis of the strawberry pomace fraction (PF) (A), water-soluble fraction (WSF) (B), water-insoluble fraction (WIF) (C), and fractions microwave-extracted for 3 min, 120 °C, pH 1.0 (D) and 3 min, 120 °C, pH 2.0 (E). HPSEC detectors were light scattering at 90 °C , differential pressure viscometer , refractive index  and ultraviolet absorption at 280 nm..

Strawberry PF, WSF, WIF, and MWE at pH values 1.0 and 2.0 and extraction times 3, 6 and 10 min were analyzed for its weight-average molar mass (*M*_*w*_), weight-average intrinsic viscosity (*η*_*w*_), size (radius of gyration, *R*_*gz*_) and shape (Mark-Howink, *M-H (α)*) (Fig. 4, Table 5). The polydispersity (*M*_*w*_/*M*_*n*_) (11.1–26.4) of PF, WSF and WIF had a much broader distribution as compared to MWE (1.37–4.77). The *M*_*w*_ distribution ranged from 7.94 to 2376 kDa, *η*_*w*_ 0.29–3.75 dL/g and *R*_*gz*_ 31.5–65.2 nm as shown in Table 5. Strawberry MWE at 10 min, 80 °C had the highest *Mw* (2376 kDa) and *η*_*w*_ (3.75 dL/g) when compared to the same microwave extraction conditions used with red beet fiber (*Mw* of 1036 kDa and *η*_*w*_ 3.71 dL/g) [22] and blueberry fiber (*Mw* of 1072 kDa and *η*_*w*_ 2.80 dL/g) [21]. For strawberry MWE at 6 min, 120 °C, 1st fraction had a *Mw* of 775 kDa with *η*_*w*_ of 1.34 dL/g, while the 6 min, 120 °C, 2nd fraction had a *Mw* of 651 kDa and *η*_*w*_ 0.827 dL/g, showing a decrease in *Mw* and *η*_*w*_ by 16 % and 38 % for the second precipitation, respectively. Broxterman & Schols [25] reported that polygalacturonase-treated insoluble strawberry pectin and cellulose had a HPSEC Mw peak at approximately 80 kDa, while the arabinase-treated fraction had a *Mw* peak at approximately 5 kDa. While pectin was not the only polysaccharide in our strawberry MWE fractions, all the fractions extracted at pH 2 had higher *Mw* than 80 kDa (Table 5). Pose et al. [3] showed that strawberry pectin size exclusion chromatograms shifted to higher molecular mass when strawberry polygalacturonase and pectate lyase genes were silenced. The pectin *Mw*, *η*_*w*_ and *R*_*gz*_ depended on the tissue sources and what conditions were used to extract it [[21], [36], [69], [70]].

**Table 5:** Polydispersity, molar mass, intrinsic viscosity, radius of gyration, hydrodynamic radius and Mark-Houwink constant of strawberry pomace fractions (mean ± standard deviation).

Extraction	*M*_*w*_*/M*_*n*_	*M*_*w*_ × 10^−3^ kDa	*η*_*w*_ (dL/g)	*R*_*gz*_ (nm)	*R*_*hzv*_ (nm)	*M-H (α)*
PF	14.2 ± 0.20	249 ± 41	0.452 ± 0.080	65.2 ± 14	45.2 ± 8.0	0.761 ± 0.20
WSF	11.1 ± 0.80	9 ± 2	0.029 ± 0.004	48.2 ± 6	34.2 ± 1.0	0.389 ± 0.20
WIF	26.4 ± 3.00	130 ± 18	0.082 ± 0.002	48.0 ± 2	56.4 ± 13.0	0.865 ± 0.10
3/120a	3.50 ± 0.02	65 ± 2	0.314 ± 0.002	31.5 ± 7	35.1 ± 2.0	0.597 ± 0.02
3/120	3.00 ± 0.20	1746 ± 34	3.040 ± 0.008	40.0 ± 1	50.7 ± 0.5	0.588 ± 0.10
6/120 Frac 1	4.77 ± 0.08	775 ± 12	1.340 ± 0.002	54.1 ± 2	59.4 ± 0.6	0.779 ± 0.20
6/120 Frac 2	4.50 ± 0.08	651 ± 9	0.827 ± 0.002	55.7 ± 4	57.9 ± 0.1	0.659 ± 0.04
10/80	1.37 ± 0.07	2376 ± 42	3.750 ± 0.040	40.3 ± 2	42.7 ± 0.4	1.250 ± 0.20

Pomace fraction (PF), water-soluble fraction (WSF), water-insoluble fraction (WIF) and fractions microwave-extracted for 3 min, 120 °C at pH 1 (3/120a), and the remaining fractions were microwave-extracted at pH 2 for 3 min, 120 °C (3/120), 6 min, 120 °C, 1st fraction collected after precipitation (6/120 Frac 1), 6 min, 120 °C, 2nd fraction collected after precipitation (6/120 Frac 2), and 10 min, 80 °C (10/80).

The *M-H (α*) constant in Table 5 showed that WSF had a compact sphere and MWE 10 min, 80 °C was an extended rod, while the other microwave-extracted fractions (PF, WIF, 3/120a, 3/120, 6/120 Frac 1, and 6/120 Frac 2) exhibited random coils. Our previously published data showed that blueberry fiber had similar random coil M − H constant values that was reported to promote anthocyanin binding [21,71]. Red beet and carrot fiber exhibited a more compact spherical shape, while random coil and linear shapes were produced following enzymatic treatment of the carrot fiber [22,24].

When HPSEC chromatograms of 3 min, 120 °C were extracted at pH 1 (Fig. 4D) and pH 2 (Fig. 4E), we saw a decrease in the Mw (∼97 %) and η_w_ (∼90 %), but not so much in R_*gz*_ (∼26 %) at pH 1 (Table 5, Fig. 4). We also observed similar trends of decreasing Mw, η_w_ and *R*_*gz*_ for red beet and blueberry pectin microwave extracted at pH 1 compared to pH 2 [[21], [22]]. As the Mw dropped with the more acidic microwave extraction conditions, the protein peak also shifted to lower molar mass (∼26.0–29.0 mL, Fig. 4). The protein associations with multiple strawberry polysaccharides present were further illustrated in Fig. S2 with the protein peak eluting between ∼18.0 mL and 33.0 mL, spreading across the entire region from high to low Mw. It is worth noting that while the MWE 10/80 min/°C fraction had the highest Mw, its main protein peak eluted at much lower molar mass (Fig. S2), which suggested little protein association with polysaccharides in this extended rod-shaped fraction.

### 3.8 NMR analysis of molecular fine structures

**Fig. 5:**
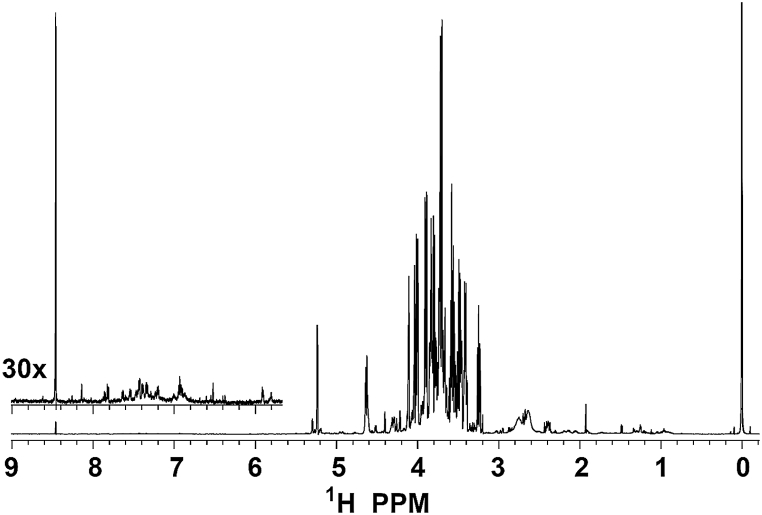
Full proton NMR spectrum with WET water suppression of strawberry PF, with inset showing the presence of low concentration anthocyanins and aromatics.

NMR spectroscopy was performed to confirm the fine structures of strawberry fractions based on our other analysis. In the strawberry PF ID ^1^H spectrum, a weak resonance was observed at 8.46 ppm, as well as a series of very weak peaks in the range of 5.8–8.2 ppm (Fig. 5). These resonances confirmed the presence of anthocyanins and aromatics at low concentrations similar to what we observed in blueberry PF [21]. Multiple groups of weak, sharp peaks, as well as several broad peaks were observed between 2.3 and 3.0 ppm. A weak rhamnose methyl doublet was observed at 1.476 ppm in the ^1^H spectrum. In the HSQC (Fig. S3), a weak methoxy group was observed at 57.0/3.202 ppm (^13^C/^1^H), as was an acetyl group with resonances at 181.9 ppm (carbonyl) and 25.64/1.935 ppm (methyl). Inspection of the strawberry PF 1D^13^C spectrum revealed four prominent peaks at 104.4, 100.9, 98.8 and 95 ppm, as well as several weaker ones at 187.2, 183.4, 107.3, 103.9, 99.6, 99.5, 99.3, 98.9, 97.6, 95.4, 95.2, 95.11 ppm, and several overlapping resonances at 95.06 ppm (Fig. S4, Table S1). The resonances at 107.3, 104.4 and 100.9 ppm were assigned as the fructose C2 of α-furanose, β-furanose and β-pyranose, respectively, thereby confirming the presence of this free sugar in our strawberry PF.

**Fig. 6:**
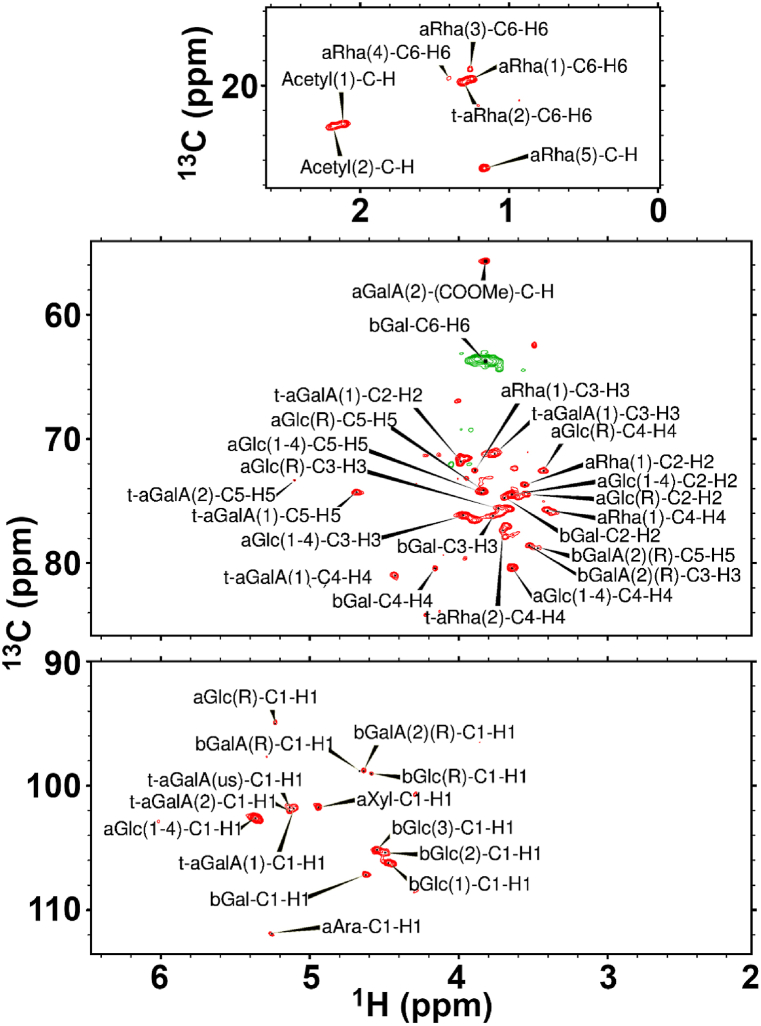
The fully-labelled multiplicity edited, sensitivity enhanced HSQC of strawberry 6 min, 120 °C, 2nd fraction at 75 °C in which –CH– correlations appear as red peaks, and –CH_2_- correlations appear as green peaks. The “a” and “b” prefixes for each monosaccharide label refers to α and β, respectively. (For interpretation of the references to color in this figure legend, the reader is referred to the Web version of this article.)

The strawberry 6 min, 120 °C, 2nd fraction had multiple resonances in the anomeric region of the 1D ^1^H spectrum with the following chemical shifts: 5.366, 5.281, 5.270, 5.232, 5.138, 5.12 (shoulder), 5.104, 5.037, 4.976, 4.950, 4.921, 4.817, 4.764, a series of sharp peaks at 4.687–4.590, and 4.557, 4.541, 4.477, 4.429 ppm (Table 6). However, no clearly discernible coupling could be measured. A series of presumed acetyl groups were observed between 2.08 and 2.22 ppm, with an uncharacteristically sharp resonance at 2.01 ppm (Fig. 6). Likewise, multiple resonances arising from Rha-C6 were observed between 1.25 and 1.36 ppm (data not shown). More broad resonances were indicated at about 0.9 and 0.95 ppm. As these are likely methyl groups containing three protons each, and as they contribute no more than 4 % to the spectral intensity, their contributions to the total composition were small.

**Table 6:** Carbohydrate structures assigned from 2D NMR analysis of the strawberry pomace 6 min, 120 °C, 2nd fraction.

	C1	C2	C3	C4	C5	C6	H1	H2	H3	H4	H5	H6	CO_2_CH_3_	CO_2_CH_3_
αAra	111.8	–	–	–	–	–	5.254	–	–	–	–	–	–	–
αGlc(1 → 4)	102.6	74.46	76.18	80.31	74.29	–	5.367	3.642	3.966	3.638	3.842	–	–	–
αGlc(R)	94.99	74.46	75.61	72.53	74.17	–	5.23	3.547	3.734	3.431	3.831	–	–	–
t-αGalA(1)	101.8	71.83	71.18	81	74.23	–	5.104	3.986	3.752	4.428	4.681	–	–	–
t-αGalA(2)	102	–	–	–	73.36	–	5.152	4.011	–	–	5.094	–	55.68	3.822
t-αGalA(us)	101.8	–	–	–	–	–	5.132	–	–	–	–	–	–	–
βGal	107.2	74.77	76.19	80.45	–	63.8	4.625	3.699	3.765	4.161	–	3.819	–	–
βGlc(1)	106.2	–	–	–	–	–	4.47	3.571	–	–	–	–	–	–
βGlc(2)	105.4	73.62	–	–	–	–	4.486	–	–	–	–	–	–	–
βGlc(3)	105.1	–	–	–	–	–	4.549	3.589	–	–	–	–	–	–
βGlc(R)	99.03	–	–	–	–	–	4.594	3.523	–	–	–	–	–	–
βGalA(1)(R)	98.76	–	–	–	–	–	4.669	–	–	–	–	–	–	–
βGalA(2)(R)	98.8	–	78.57	–	78.74	–	4.639	3.258	3.524	–	3.463	–	–	–
αXyl	101.7	74.41	–	–	–	–	4.945	3.571	3.728	–	–	–	–	–
αRha(1)	–	73.68	72.51	75.71	–	19.44	–	3.556	3.889	3.414	–	1.25	–	–
t-αRha(2)	–	–	–	77.44	–	19.74	–	–	–	3.705	–	1.304	–	–
αRha(3)	–	–	–	–	–	18.69	–	–	–	–	–	1.26	–	–
αRha(4)	–	–	–	–	–	19.41	–	–	–	–	–	1.402	–	–
αRha(5)	–	–	–	–	–	26.65	–	–	–	–	–	1.166	–	–

Arabinose (Ara), fructose (Fru), galactose (Gal), galacturonic acid (GalA), glucose (Glc), glucuronic acid (GlcA), fucose (Fuc), mannose (Man), rhamnose (Rha), xylose (Xyl), alpha (α), beta (β), terminal (t), reducing end (R), 4,5-unsaturated (us). The parenthetical numerals are for identification purposes only, and do not refer to locations in the polysaccharide(s), except for (1 → 4). their respective carbonyls at 176.4 and 176.1 ppm (Fig. 6).

The ^13^C spectrum of the strawberry 6 min, 120 °C, 2nd fraction contained anomeric peaks at 111.9, 107.2, 106.2, 105.5, 105.2, 103.5, 102.8, 102.5, 101.8, 98.8, 97.8, 95.3, and 95 ppm (Table 6). Other notable resonances include a very small methoxy peak at 55.6 ppm, a peculiarly sharp peak at 26.6 ppm, a very weak and likely acetyl methyl at 23.1 ppm, and three Rha-C6 at 19.5, 18.7 and 14.5 ppm. In addition, a peak was observed at 187.4 from a possible aldehyde and a series of small overlapping peaks at 171.9, 176.1 and 173.5 ppm from carboxylic acid carbons. 2D NMR analysis (Fig. 6) indicated the presence of both α- and β-galacturonic acid, which was consistent with a homogalacturonan reducing end, α- and β-glucose, α-arabinose, α-xylose and α-rhamnose (Fig. 6). Comparison of these β-Glc-C1-H1resonances to those found in oat β-glucan [72], confirmed that these strawberry resonances are from β-glucan, with each belonging to a different linkage. The β-Glc(1) corresponded to their G4 assignment at the non-reducing end of the sequence tetrasaccharide **G4**G4G3G, while β-Glc(2) was at the non-reducing end in trisaccharide **G4**G3G, and Glc(3) was the 4G4 in tetrasaccharide G**4G4**G3. In addition, the 1D-1H spectrum contained some small peaks at ∼4.75 ppm, which could correspond to the fragments they identified as G**4G3**G and G4G**4G3**G. A methyl ester was observed at 55.77/3.824 ppm, and two acetyl groups are detected with methyl resonances at 23.06/2.114 and 23.3/2.181 ppm, with.their respective carbonyls at 176.4 and 176.1 ppm (Fig. 6).

For comparison, the strawberry 3 min, 120 °C, pH1 microwave extracted fraction was repeated in 2023 producing a fraction that was similar to that produced in 2019 except that the molecular weight was lower (7.94 kDa in 2023 vs 65 kDa in 2019). The 1D ^1^H NMR analysis of this 2023 3 min, 120 °C, pH1 fraction had an anomeric region with its strongest resonances at 5.080 ppm and 4.732 ppm, which were assigned as the anomeric and H5 resonances of a terminal α-GalA residue, respectively (Table S2), confirming the galacturonic acid-rich composition. The H5 resonance from another terminal αGalA was also seen in this region (5.118 ppm), which appeared to be shifted due to methyl esterification (Fig. S3). The methyl ester was observed at 3.8/55.7 ppm (^1^H/^13^C) in the HSQC (Fig. S3), and correlations to it through its C6 were seen in the HMBC (data not shown). Three acetylation resonances were observed between 2.03 and 2.22 ppm, although the 2D NMR could not identify the residues associated with them despite the low molecular weight of this fraction (Fig. S5). At least seven methyl groups were detected between 1.05 and 1.30 ppm, with the most intense at 1.16 ppm, which are likely all rhamnose C6, although some of the lower intensity ones may have been from fucose. The 1D^13^C spectrum displayed carboxylic acid peaks at 177.8 and 185 ppm (data not shown), the former of which was moderately strong, the latter weak, as well as a weak ester resonance at 173.7 ppm. In the anomeric region, a weak resonance was seen at ∼108 ppm, which is likely an αAra, but it could not be detected in the 2D spectra (Fig. S5). The strongest anomeric carbons were assigned as being from several αGalA and were observed at ∼102 ppm (Fig. S5), further confirming the galacturonic acid-rich composition of this fraction. The methyl carbon of the ester was seen as a medium intense resonance at 55.7 ppm in the 1D^13^C spectrum. What appeared to be a very strong acetyl methyl resonance was observed at 26.7 ppm, but the HSQC spectrum indicated that this is a rhamnose methyl detected at 1.16/26.7 ppm. Acetyl methyl resonances were at 2.09/23.2 ppm (Fig. S5), and its carbonyl was at 176.4 ppm, according to the HMBC (data not shown). Overall, the NMR analysis of the strawberry 3 min, 120 °C, pH1 fraction indicated that the dominant monosaccharide was galacturonic acid from homogalacturonan. There were also weak indications of α- and β-glucose, α-xylose, α-rhamnose and α-arabinose, which was consistent with the previous data supporting minor amounts of xyloglucan, β-glucan and rhamnogalacturonan I in this fraction.

## 4 Conclusions

The strawberry pomace described in this paper was an insoluble dietary fiber consisting of fucogalacto-xyloglucan, methyl-esterified rhamnogalacturonan I and II pectin with arabinogalacto-oligosaccharides, β-glucan, xylan, glucomannan, protein, fat, and anthocyanins. The arabino- and galactose-oligosaccharide structures in strawberry pomace are known to confer prebiotic activity, while the strawberry xyloglucan associated with pectic oligosaccharides also was previously reported with prebiotic activity as well as pathogen anti-adhesion activity, and the strawberry β-glucan had similar structure to oat β-glucan soluble fiber that was previously reported to have cholesterol-lowering activity. These are the first reports of these structures with potential bioactivity in commercial strawberry pomace fiber. Therefore, strawberry pomace can be a multiple-function bioactive food ingredient and thickening agent with relatively high levels of protein and antioxidant anthocyanins based on the molar mass, viscosity, and random coil shape of fiber polysaccharides.

## Funding

This work was supported through a Material Transfer Research Agreement (58-8072-8-002) between the USDA and Ingredion Inc. The glycosyl-linkage analysis was supported in part by the U.S. Department of Energy, Office of Science, Basic Energy Sciences, grant number DE-SC0015662 to Parastoo Azadi at the Complex Carbohydrate Research Center, University of Georgia. The balance of the funding was from National Program 306, Dairy and Functional Foods Research Unit, 10.13039/100020664USDA, ARS, ERRC Project 8072-41000-109 “New Bioactive Dairy Products for Health-Promoting Functional Foods”.

## Data availability

Data will be made available on request.

## CRediT authorship contribution statement

**Arland T. Hotchkiss:** Writing – review & editing, Writing – original draft, Supervision, Methodology, Investigation, Funding acquisition, Conceptualization. **Hoa K. Chau:** Writing – review & editing, Writing – original draft, Visualization, Methodology, Investigation, Data curation. **Gary D. Strahan:** Writing – review & editing, Writing – original draft, Methodology, Investigation. **Alberto Nuñez:** Methodology, Investigation. **Andrew Harron:** Investigation. **Stefanie Simon:** Methodology, Investigation. **Andre K. White:** Methodology, Investigation. **Senghane Dieng:** Methodology, Investigation. **Eugene R. Heuberger:** Writing – original draft, Methodology. **Ian Black:** Writing – original draft, Methodology, Investigation. **Madhav P. Yadav:** Writing – review & editing, Writing – original draft, Supervision. **Marjorie A. Welchoff:** Writing – review & editing, Writing – original draft, Methodology, Investigation, Conceptualization. **Julie Hirsch:** Writing – review & editing, Writing – original draft, Methodology, Investigation, Funding acquisition, Conceptualization.

## Declaration of competing interest

The authors declare no conflict of interest.
