# Supplementary material for: Structural characterization of strawberry pomace
Source: Heliyon. 2024 Apr 17;10(9):e29787. doi: 10.1016/j.heliyon.2024.e29787 (PMC11066319; doi:10.1016/j.heliyon.2024.e29787)
Supplement: Multimedia component 1 [file mmc1.docx]

Appendix.

Supplementary Material

**Fig. S1.** Total dietary fiber (TDF), soluble dietary fiber (SDF) and insoluble dietary fiber (IDF) weight percent (%) mean values of duplicate analysis are shown for the strawberry pomace fraction (PF), water-soluble fraction (WSF) and water-insoluble fraction (WIF).


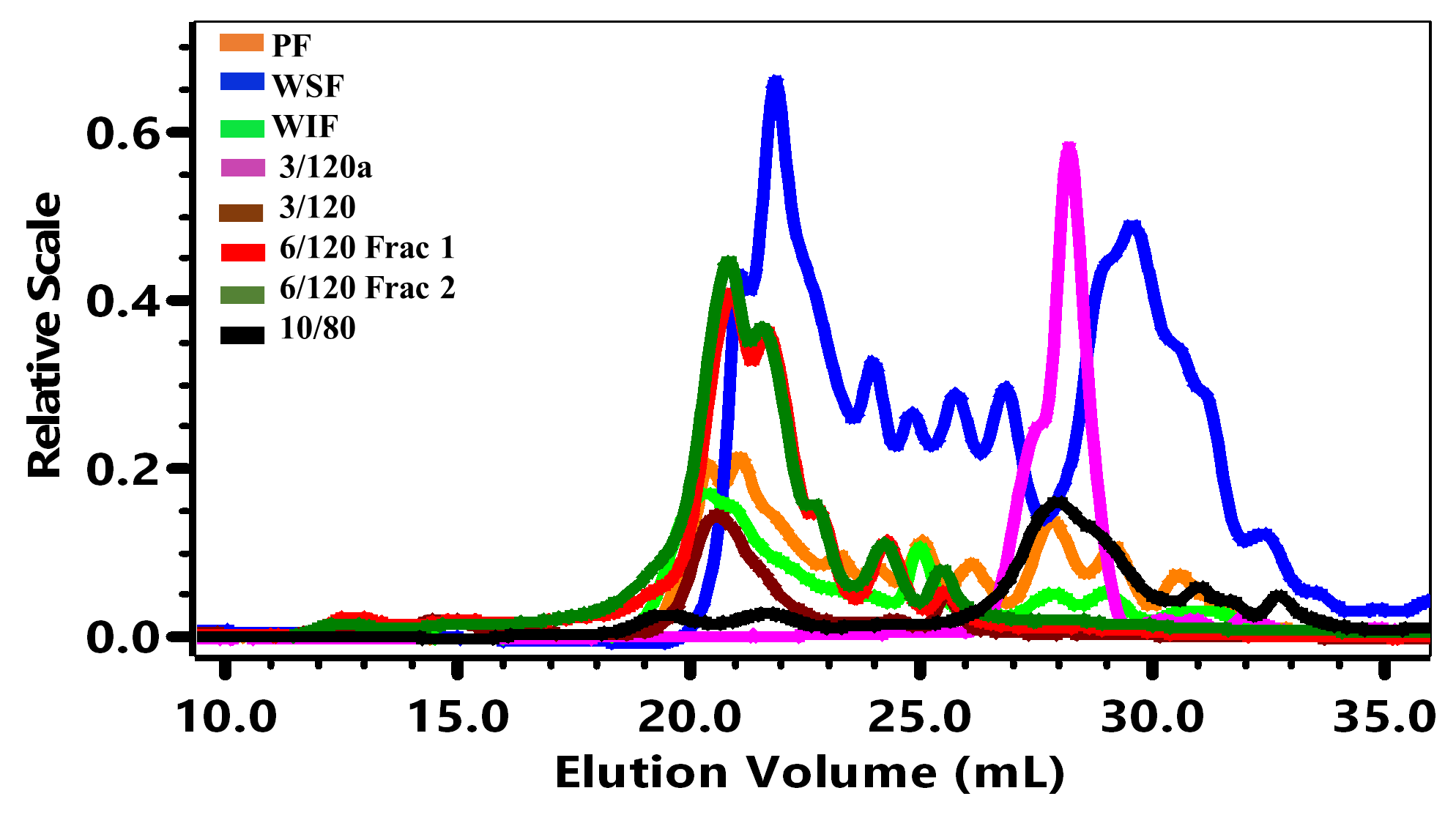


**Fig. S2.** Superimposed UV chromatograms of PF, WSF, WIF, MWE 3/120a (pH 1.0), MWE 3/120 min/˚C, MWE 6/120 min/˚C, MWEP 6/120 min/˚C and MWE 10/80 min/˚C (pH 2.0 for latter four samples).


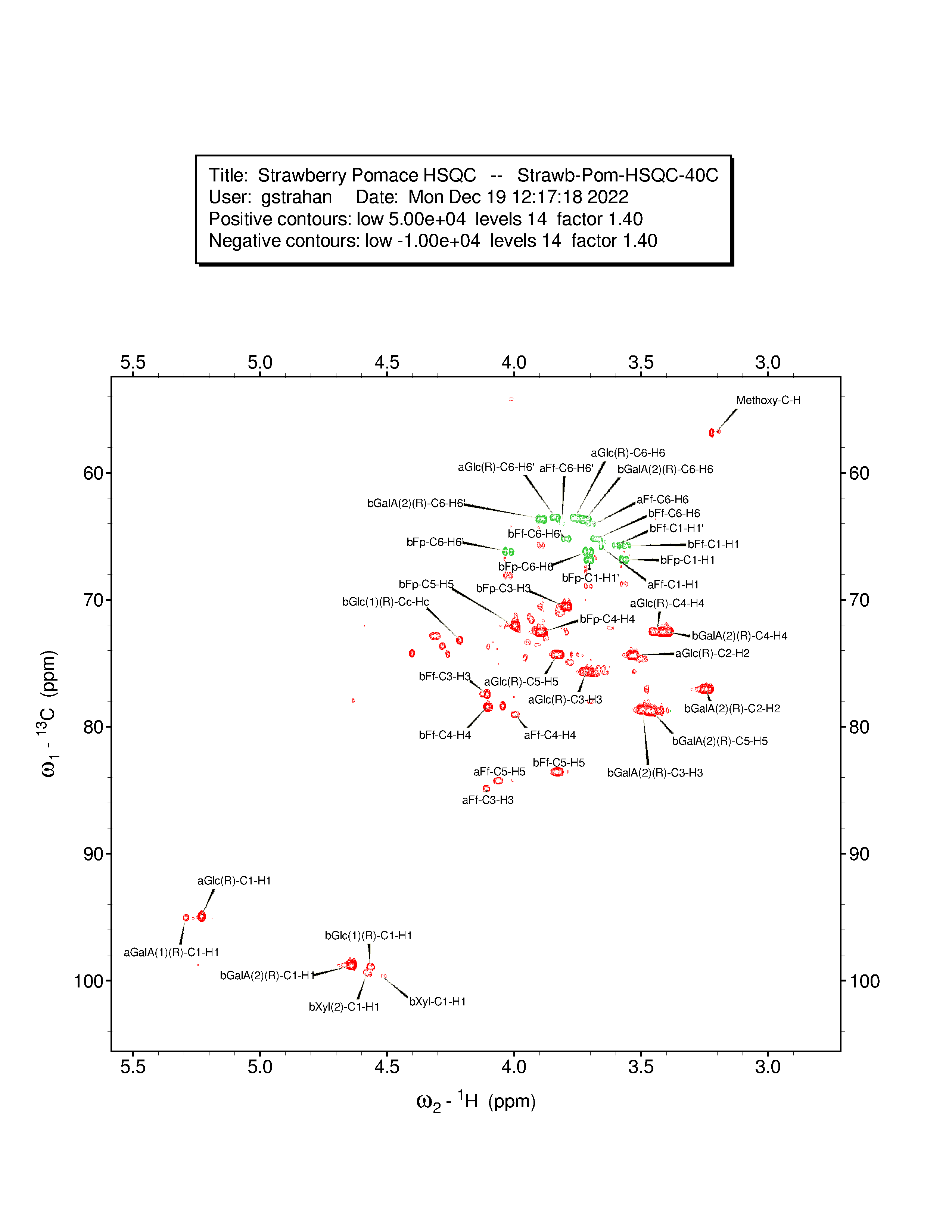


**Fig. S3.** The 2D HSQC NMR spectrum of strawberry pomace fraction PF at 75 °C in which

-CH- correlations appear as red peaks, and -CH_2_- correlations appear as green peaks. The parenthetical numerals are for identification purposes only, and do not have structural relevance.


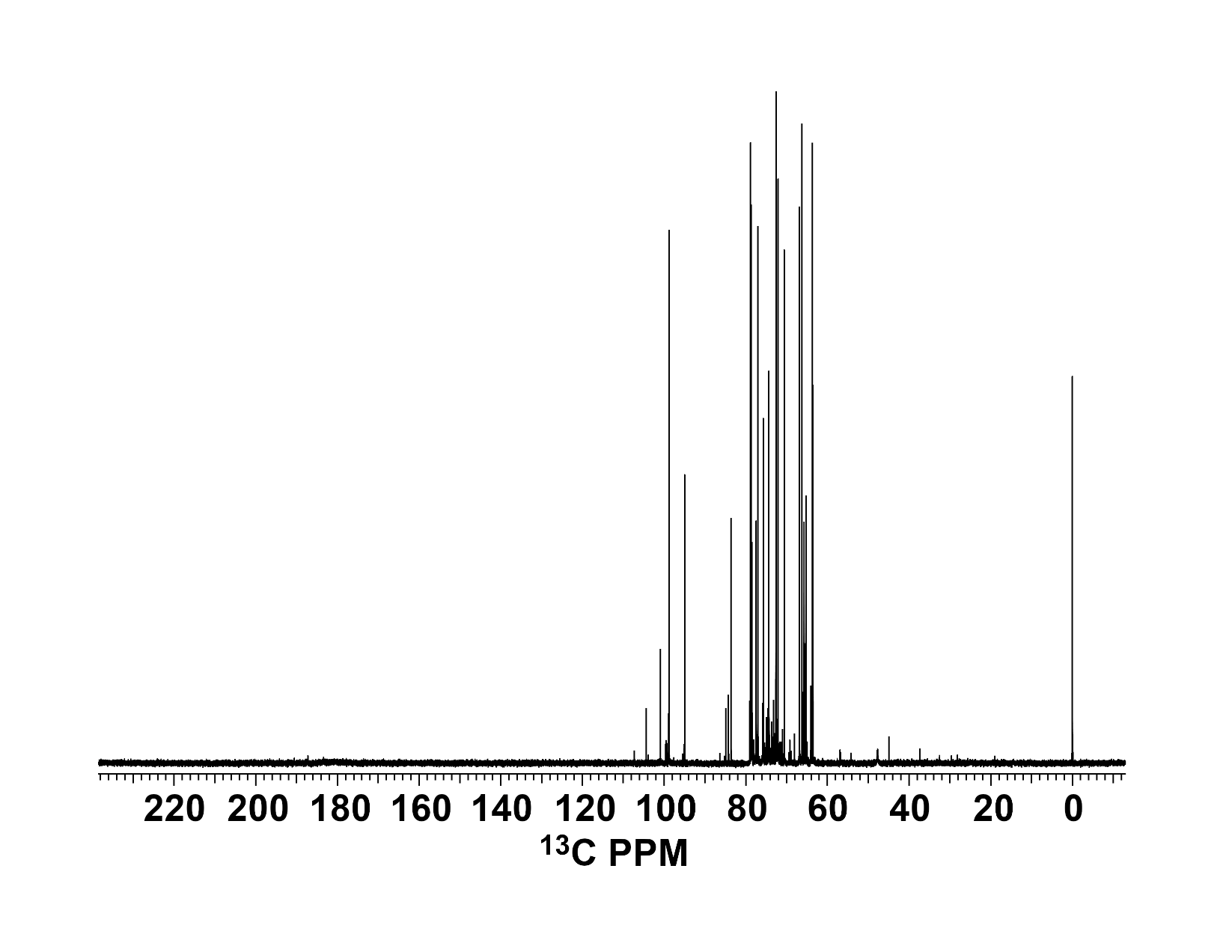


**Fig. S4.** Full carbon NMR spectrum of strawberry pomace fraction PF at 75 °C.

**Table S1**

NMR chemical shifts of the strawberry pomace fraction PF.

|  | *J*(H1/H2) | C1 | C2 | C3 | C4 | C5 | C6 | H1 | H2 | H3 | H4 | H5 | H6 |
| --- | --- | --- | --- | --- | --- | --- | --- | --- | --- | --- | --- | --- | --- |
| α-Rha | - | - | - | - | - | - | 18.99 | - | - | - | - | - | 1.369 |
| α-Fru*f* | - | 65.85 | 107.3 | 84.98 | 79.26 | 84.42 | 64.11 | 3.65 | - | 4.108 | 3.993 | 4.069 | 3.696, 3.805 |
| α-Glc(R) | 3.7 Hz | 94.97 | 74.39 | 75.7 | 72.66 | 74.35 | 63.64 | 5.228 | 3.533 | 3.715 | 3.414 | 3.827 | 3.754, 3.838 |
| β-Glc(1)(R) | 7.4 Hz | 98.96 |  | (73.21), (75.86, 74.68) | | | | 4.562 | (4.035), (3.678, 3.817, 4.215, 3.672, 3.499) | | | | |
| β-Fru*f* | - | 65.63 | 104.4 | 77.58 | 78.52 | 83.61 | 65.22 | 3.557, 3.608 |  | 4.104 | 4.10 | 3.828 | 3.678, 3.787 |
| β-Fru*p* | - | 66.95 | 100.9 | 70.72 | 72.58 | 72.05 | 66.22 | 3.569, 3.700 |  | 3.798 | 3.886 | 3.995 | 3.712, 4.021 |
| β-Xyl | 7.6 Hz | 99.71 | - | - | - | - | - | 4.508 | 3.506 | 3.584 | 3.654 | 3.929 | - |
| β-Xyl(2) | - | 99.44 | - | - | - | - | - | 4.573 | - | - | 3.632 | 3.93 | - |
| α-GalA(1)(R) | 3.6 Hz | 95.06 | 72.23 | 74.23 |  |  | 177.7 | 5.292 | 3.817 | - | (3.904, 4.282, 4.393) | | |
| β-GalA(2)(R) | 7.8 Hz | 98.83 | 77.12 | 78.71 | 72.62 | 78.79 | 63.77 | 4.634 | 3.243 | 3.490 | 3.406 | 3.455 | 3.717, 3.885 |

The parenthetical numerals are for identification purposes only, and do not have structural relevance.


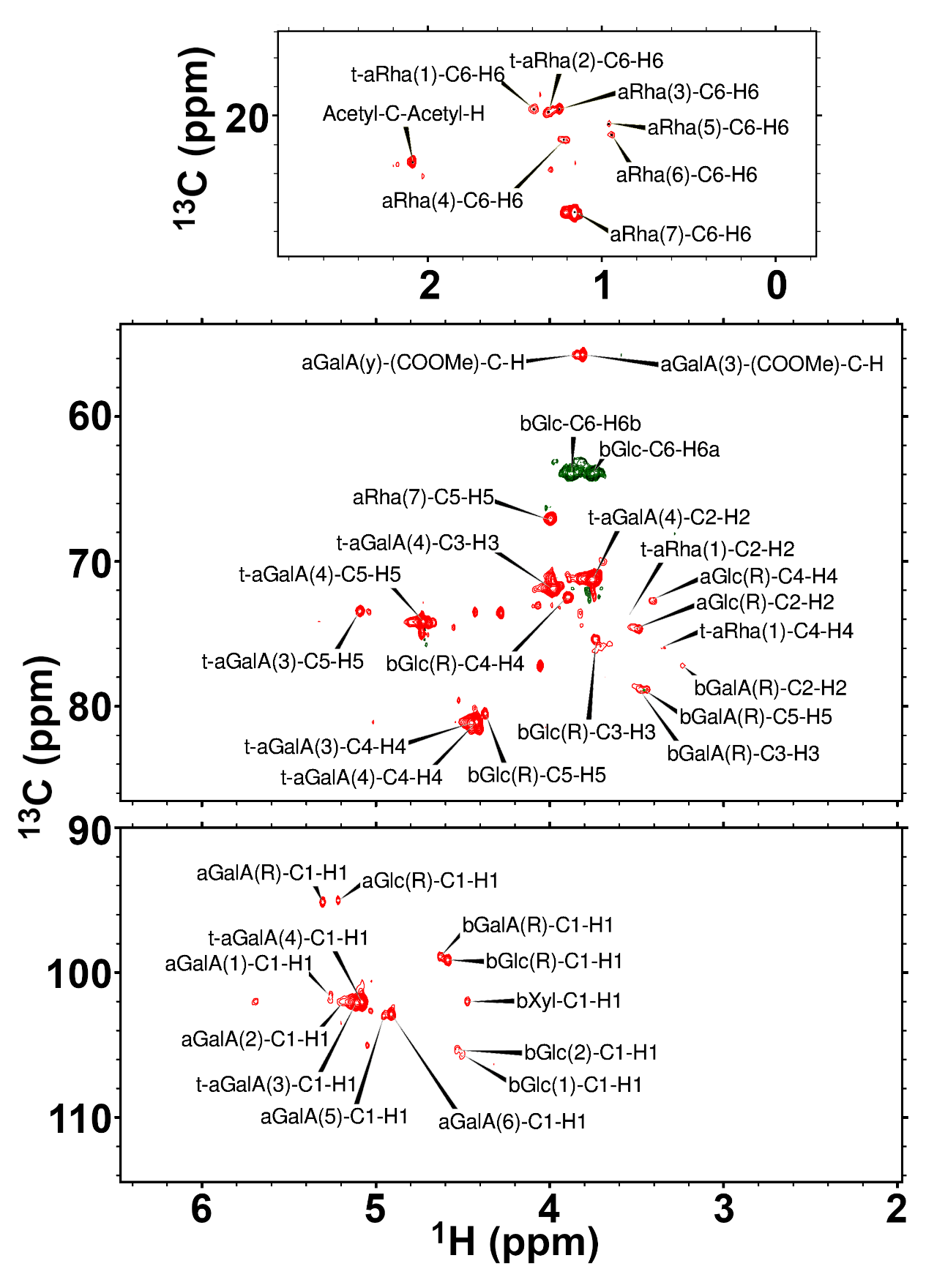


**Fig. S5.** The complete HSQC spectrum of strawberry 3 min, 120 ˚C, pH1 fraction, measured at 70 ˚C in which -CH- correlations appear as red peaks, and -CH_2_- correlations appear as green peaks. Here an “a” preceding the residue abbreviation refers to the α conformation, while “b” refers to the β conformation.

**Table S2**

Strawberry 3 min, 120 ˚C, pH1 assignments based on 2D NMR analysis.

|  | C1 | C2 | C3 | C4 | C5 | C6 | H1 | H2 | H3 | H4 | H5 | H6 | COO**C**H_3_ | COOC**H_3_** |
| --- | --- | --- | --- | --- | --- | --- | --- | --- | --- | --- | --- | --- | --- | --- |
| αGalA(1) | 101.6 | - | - | - | - | - | 5.262 | - | - | - | - | - | - | - |
| αGalA(2) | 102 | - | - | - | - | - | 5.184 | - | - | - | - | - | - | - |
| t‑αGalA(3) | 102.1 | - | - | 81.13 | 73.46 | - | 5.112 | 3.757 | 3.983 | 4.449 | 5.091 | - | 55.76 | 3.808 |
| t‑αGalA(4) | 102 | 71.26 | 71.89 | 81.08 | 74.21 | 177.6 | 5.082 | 3.755 | 3.982 | 4.422 | 4.722 | - | - | - |
| αGalA(5) | 103 | - | - | - | - | - | 4.953 | - | - | - | - | - | - | - |
| αGalA(6) | 102.9 | - | - | - | - | - | 4.911 | 3.738 | 4.378 | 3.958 | | | - | - |
| αGalA(R) | 95.1 | - | - | - | - | - | 5.305 | 3.821 | 3.988 | (3.976, 4.42) | | | - | - |
| αGalA(y) | - | - | - | - | - | 173.6 | - | - | - | - | - | - | 55.75 | 3.847 |
| αGlc(R) | 95.03 | 74.65 | - | 72.62 | - | - | 5.22 | 3.53 | - | 3.411 | - | - | - | - |
| t‑αRha(1) | - | 73.77 | - | 75.89 | - | 19.53 | - | 3.547 | - | 3.354 | - | 1.391 | - | - |
| t‑αRha(2) | - | - | - | - | - | 19.71 | - | - | - | - | 3.837 | 1.298 | - | - |
| αRha(3) | - | - | - | 75.03 | 71.97 | 19.53 | (3..394, 3.88) | | | | 3.758 | 1.246 | - | - |
| αRha(4) | - | - | - | - | - | 21.64 | - | - | - | - | - | 1.205 | - | - |
| αRha(5) | - | - | - | - | - | 20.48 | - | - | - | - | - | 0.96 | - | - |
| αRha(6) | - | - | - | - | 62.27 | 21.38 | - | - | - | - | - | 0.9375 | - | - |
| αRha(7) | - | - | - | - | 67.08 | 26.65 | (3.88, 4.474) | | | | 3.997 | 1.161 | - | - |
| βGalA(R) | 98.88 | 77.18 | 78.85 | - | 78.88 | - | 4.628 | 3.236 | 3.48 | - | 3.463 | - | - | - |
| βGlc(1) | 105.7 | - | - | - | - | 63.93 | 4.504 | 3.318 | 3.497 | | | 3.754, 3.874 | - | - |
| βGlc(2) | 105.4 | - | - | - | - | - | 4.53 | 3.365 | 3.655 | | | | - | - |
| βGlc(R) | 99.12 | 71.25 | 75.43 | 72.48 | 80.53 | - | 4.586 | 3.49 | 3.734 | 3.898 | 4.321 | - | - | - |
| βXyl | 102 | - | - | - | - | - | 4.478 | 3.477 | - | - | - | - | - | - |
